# A smiling mouth expression modulates gaze appearance by enhancing how the eyes light up

**DOI:** 10.1038/s41598-026-56132-1

**Published:** 2026-06-10

**Authors:** Giulia Parovel, Stefano Guidi

**Affiliations:** Department of Social, Political and Cognitive Sciences, University of Siena, Siena, Italy

**Keywords:** Composite facial expressions, Visual illusions, Face illusions, The mouth-eye brightening effect, Perceptual organization, Mona Lisa, Gaze perception, Psychology, Human behaviour

## Abstract

Research on composite facial expressions has shown that a smiling mouth, when combined with neutral eyes, can influence how the eyes are interpreted. From a gestalt-oriented perspective, we propose that smiling not only affects emotion recognition but also produces a fascinating, compelling visual counterpart: an enhancement of the eyes’ apparent luminosity, or ‘lighting up’. We tested this hypothesis in two within-subject experiments by collecting ratings of perceived eye brightness and smile intensity for whole faces, as well as for eyes and mouths presented isolated. In Experiment 1, we combined neutral eyes with neutral, slightly smiling, and fully smiling mouths. Results show that, for each stimulus, eye brightness ratings increased as a function of smile intensity. In Experiment 2, we combined neutral and Duchenne-smiling eyes with neutral and smiling mouths. Data analysis reveals that a smiling mouth significantly enhances the luminosity ratings of Duchenne-smiling eyes compared to their own perceptual baseline (individual eyes presented alone). Furthermore, a neutral mouth appears to ‘dim’ the brightness of Duchenne-smiling eyes. Through further pictorial demonstrations, we suggest that this phenomenon is remarkably robust, occurring in naturalistic photographic portraits, pareidolic faces and paintings. We propose that this subtle yet compelling effect represents a perceptual signature of the gaze, intensifying facial aliveness as the mouth modulates the eyes’ appearance. Mirroring the relational logic of the Wollaston illusion, our findings demonstrate that the eyes and mouth operate in synergy, forming a unified expression where the visual system integrates individual features into a harmonious and living whole.

## Introduction

Smiling, as is generally experienced, is not limited to the mouth area, but involves the whole face. When a person smiles genuinely, the face appears more attractive and healthier; also, smiling eyes appear brighter and alive, and the corners of the eyes are wrinkled (the so-called Duchenne smile). Sometimes, however, the mouth and eyes can each play a different role: for example, we can smile with the mouth only, keeping the upper part of the face neutral, as it happens when people smile without really being happy, just out of courtesy (namely, non-Duchenne smile). As it is well known, people displaying a Duchenne smile are perceived as more positive (i.e. authentic, genuine, real, attractive and trustworthy) than those displaying a non-Duchenne smile^1,2^).

**Fig. 1:**
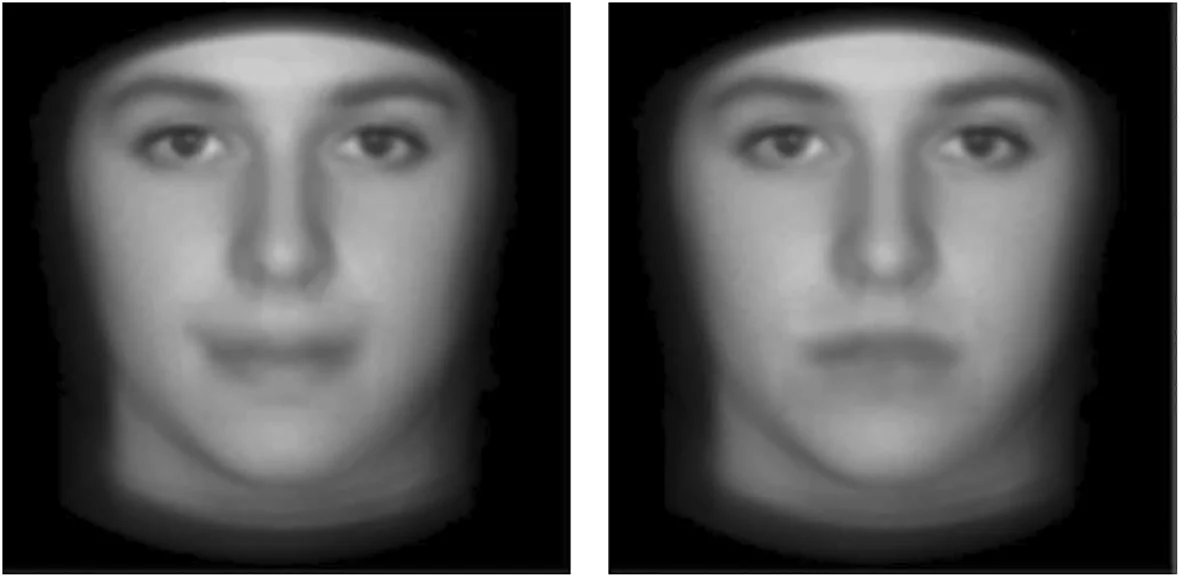
Modifying the mouth alone, the eyes appear to ‘light up’ with the smile. *Source:* Mangini & Biederman, 2004.

Previous research on composite facial expression has mainly been concerned with observers’ accuracy in the identification of emotional expressions and in resolving incongruences between mouth and eye expressions in composite faces. Taking a different perspective, inspired by phenomenological observations and by Mangini and Biederman’s demonstration^3^ (Fig. 1), we propose that altering the mouth to a smile affects not only emotion recognition, but also the perceived brightness and vividness of the eyes. Specifically, we hypothesise that neutral eyes may appear to ‘light up’ when combined with a smiling mouth, and vice versa: smiling, luminous eyes may appear less bright when combined with a neutral mouth.

### Evidence of mouth-eye interaction in facial expression processing

Experimental evidence concerning the mouth-eye interaction was first provided in the research context of facial emotion recognition.

Since Dunlap’s work^4^, using the *composite face paradigm* (e.g., a smiling top face with an angry bottom face), it has been clear that the lower region of the face plays a critical role in recognising certain expressions, particularly positive ones (see also^5–7^).

Calder and colleagues^8^ largely confirmed these previous findings, showing that expressions such as anger, fear, and sadness rely more on the upper face, whereas happiness and disgust depend more on the lower face. Moreover, they have shown that mismatched facial halves strongly interfere with emotion identification, supporting a configural model of facial expression recognition in which upper and lower halves combine into a new holistic expression (see also^9^).

Calvo et al.^10^ presented identical eyes combined with congruent or incongruent mouths (smiling, angry, or sad) or with no mouth. They found that a congruent mouth facilitates the recognition of the eye expression, whereas a non-congruent mouth greatly interferes with it. Notably, smiling mouths “mislead viewers to interpret non-happy eyes as happy” (*p*. 1178), more than angry or sad mouths. This occurred even when only the eyes were looked at directly, suggesting that the high visual saliency of the smile would allow for more extrafoveal projection to surrounding face regions (see also^11,12^). Especially in ambiguous—i.e., internally incoherent—expressions, the smile would reveal its highly diagnostic role in emotion categorisation (see^12^).

In addition, Tanaka et al.^13^ found that happy expressions were recognized more quickly and accurately than angry expressions regardless of context. They propose that holistic processing is engaged when emotional information is ambiguous, whereas clear expressions are processed more analytically, via parts-based mechanisms.

While these works rely on the assumption of discrete basic emotion categories^14^, other studies adopt a dimensional perspective, in which expressions are mapped along valence and arousal dimensions^15–17^. Following this approach, also Kilpeläinen and Salmela^18^ found that the mouth generally carries more weight, potentially because a smiling mouth directly influences the interpretation of the eyes, and argue that neither categorical nor dimensional theories fully capture their results, and instead propose a hybrid account (see also^19^).

As an alternative perspective, Calvo and Nummenmaa^17^ propose that expression recognition is based mainly on the perceptual analysis of morphological features. According to their model, low-level perceptual processing precedes affective encoding along the valence dimension, which is then followed by semantic categorisation^20^.

Overall, face expressions can be processed using both analytical (part-based) and holistic (gestalt) strategies depending on the degree of ambiguity^21^. Happiness may be a special case with the smiling mouth allowing the fastest emotion recognition^13,17^.

**Fig. 2:**
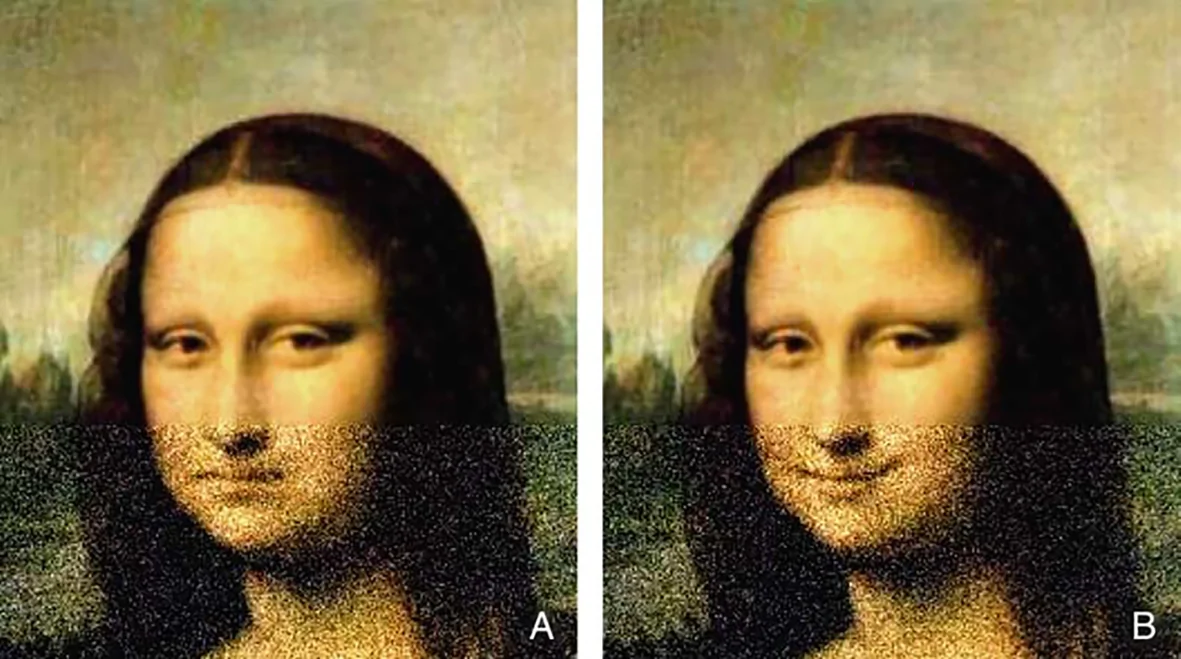
By superimposing noise to the lower part of the image Mona Lisa’s eyes look *much happier. * *Source:* Kontsevich & Tyler, 2004.

A compelling illustration of the influence of the mouth on the eyes is provided by Kontsevich and Tyler^22^, who manipulated the Mona Lisa by superimposing noise over the entire image. They found that adding noise consistent with a smiling mouth to the lower half of the face made the eyes appear happier (Fig. 2). They suggest a configural projection from the mouth region to the eyes.

In summary, while several studies have documented the powerful role of smiling in driving expression categorisation—and work on the Mona Lisa has examined how spatial features affect perceived facial ambiguity (see also^23–26^) —none of these investigations has addressed whether smiling can modulate the apparent brightness of the eyes themselves, as evident in Mangini and Biederman’s demonstration^3^.

Indeed, while the impression of the eyes ‘lighting up’ is inherently linked to the facial expression, it is not reducible to it. In principle, an observer could categorize a face as ‘smiling’ based on the mouth’s configuration—as typically occurs in composite face tasks—without necessarily experiencing a change in the appearance of the gaze. Our hypothesis, however, is that the smile does not merely provide a label for the expression, but actively intensifies a pre-existing quality of the gaze. This issue forms the basis of the present work.

### A gestalt-oriented perspective: the *faceness* principle

From a gestalt-oriented view, *faceness* acts as a grouping factor that integrates different parts into a coherent whole, where the parts are strongly interdependent. In such a system, modifying a single part affects the perception of the entire configuration, and parts may appear different depending on the whole in which they are embedded^27^.

This principle is evident in the phenomenon of *pareidolia*, the tendency to perceive faces even in inanimate configurations, as shown in the artworks of Arcimboldo (1527–1593) or natural formations such as clouds and rocks^28^. Moreover, newborns show a spontaneous preference for geometric patterns with more elements in the upper half—an early sensitivity that supports face detection (e.g.,^29–31^). These findings support the existence of hard-wired sensitivity specifically tuned to face recognition, deeply rooted in defined neural mechanisms, as it has long been recognized in several fields of scientific research and across many different methodologies^32^.

**Fig. 3:**
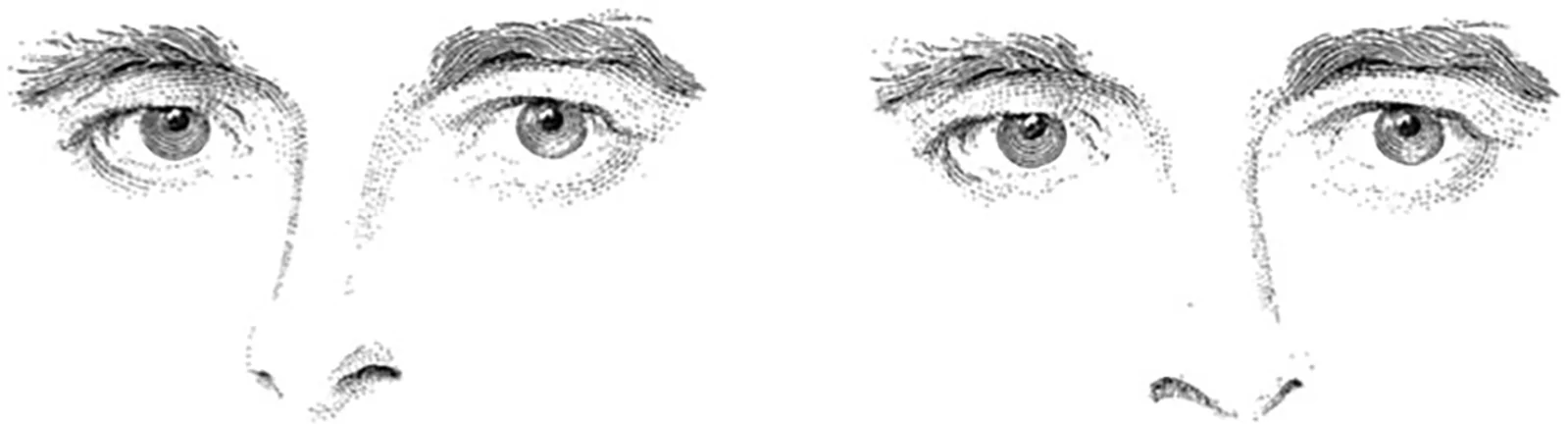
The *Wollaston effect* (1824). A change in the nose affects the perceived gaze direction. *Source:* Wollaston, 1824.

Face perception also gives rise to many illusory compelling effects, all of which reflect configural processing^33,34^. Classic examples include the *inverted face illusions*^35^, the *fat face thin illusion*^36^, the so-called *wobbling face illusion*, where two facial patterns compete to be attended to, causing continuous rapid reversals from one to the other^37,38^, and the *Wollaston effect*, observed in 1824^39^, where the nose seems to attract the gaze, even though the eye socket and eyebrows remain unchanged (Fig. 3). Changes in perceived gaze direction of up to 20° have been observed^40–42^. Even the position of the mouth can bias the direction of gaze^34^.

A further example of face illusion can be found within the *composite face* paradigm itself. Young and colleagues^43^ found that merging the upper and lower halves of famous faces (e.g., Marilyn Monroe’s upper half with Margaret Thatcher’s lower half) produces a novel identity, despite the familiarity with the two faces^43,44^.

It has also been reported by Valuch et al.^45,46^ that subtle manipulations of the mouth in photographs increase the perceived attractiveness of the faces; however, although a change in the brightness of the gaze seems to be observable in their stimuli, this effect went unnoticed and was not empirically investigated in their work.

### The present research: the role of the smile in the ‘lighting up’ of the eyes

It is commonly observed that eyes appear brighter when a person smiles. Darwin already noted this association. In his work on the expression of the emotions^47^, he described the connection between the brightness of eyes and smiling expressions, explaining it as a result of muscular tension, blood circulation and moisture in the eyes during laughter:

“A bright and sparkling eye is as characteristic of a pleased or amused state of mind, as is the retraction of the corners of the mouth and upper lip with the wrinkles thus produced. […] Under extreme laughter the eyes are too much suffused with tears to sparkle; but the moisture squeezed out of the glands during moderate laughter or smiling may aid in giving them lustre. […] Their brightness seems to be chiefly due to their tenseness, owing to the contraction of the orbicular muscles and to the pressure of the raised cheeks. …] Any cause which lowers the circulation deadens the eyes” (p. 206). Humans indeed are remarkably sensitive to subtle changes in facial features^48^.

In light of the evidence supporting the gestalt nature of *faceness*, we hypothesise that a smile does not merely influence emotion recognition but may also change the perceptual appearance of the eyes themselves.

Our motivation was strengthened by the compelling evidence displayed by the image reported by Mangini and Biederman^3^, citing Schwartz et al.^49^ (Fig. 1).

The image clearly shows that altering only the mouth—by adding a small shaded curvature -makes the eyes appear to ‘light up’, despite being physically identical across versions. Importantly, this effect occurs with a minimal, low-salience smile.

In line with the relational effects displayed in face illusions, *faceness* acts as a powerful organisational factor, where changes in one part modulate the perceptual qualities of another. Just as in the Wollaston illusion the perceived direction of gaze is altered by the location of the iris and by the head turn^41^, in the Mangini and Biederman image identical eyes appear more or less luminous depending on the expression of the mouth.

From a gestalt perspective, such illusions are not perceptual errors or misperceptions but context-dependent effects, where identical stimuli appear different when embedded in different configurations^33,50^. In this framework, vision concerns not only the information about individual properties of an object, such as colour, shape or size, but also their relations.

The visual processing of relations in perceptual illusions does exhibit specific signatures: it is rapid and automatic, cognitively impenetrable, sensitive to subtle visual parameters, and interacts with other perceptual processes (see^51–54^).

We hypothesise that if altering the mouth induces changes not only in emotional categorisation but also in the perceived brightness of the eyes, this would suggest that mouth-eye interaction is not limited to high-level cognitive inference but also operates at a perceptual level. This hypothesis is the focus of the present research.

### Experiment 1

Encouraged by the compelling evidence of the effect presented by Mangini and Biederman^3^ in a black-and-white, blurred portrait, we wondered whether the same effect could be observed in colour photographs of faces displaying varying degrees of smile salience, combined with a neutral expression of the eyes.

We therefore chose to focus on the apparent change in eye brightness as a dependent variable influenced by the mouth’s smile. Rather than classifying the eyes according to an emotional category, participants were asked to rate their perceived brightness, i.e., a visual property of the gaze. Additionally, we included an intermediate, subtle smile expression alongside the full smile and neutral mouth expressions to determine whether the apparent change in eye brightness was an ‘all-or-nothing’ categorical effect or a graded effect with varying degrees of intensity.

### Methods

#### Participants

Forty persons participated in the experiment on a voluntary basis. They were aged from 18 to 60 years (M = 30.8, SD = 11.9). 52.2% of them were women (N = 21), 42.5% were men (N = 17) and 2 did not disclose their gender. The participants were all of Italian nationality and Caucasian ethnicity. A power simulation showed that this sample size was sufficient to detect a medium sized effect with more than 95% power.

All participants were naïve to the purpose of the experiment and gave written informed consent according to the Declaration of Helsinki prior to their inclusion in the experiment. They reported to have normal or corrected-to-normal visual acuity.

#### Stimuli and apparatus

**Fig. 4:**
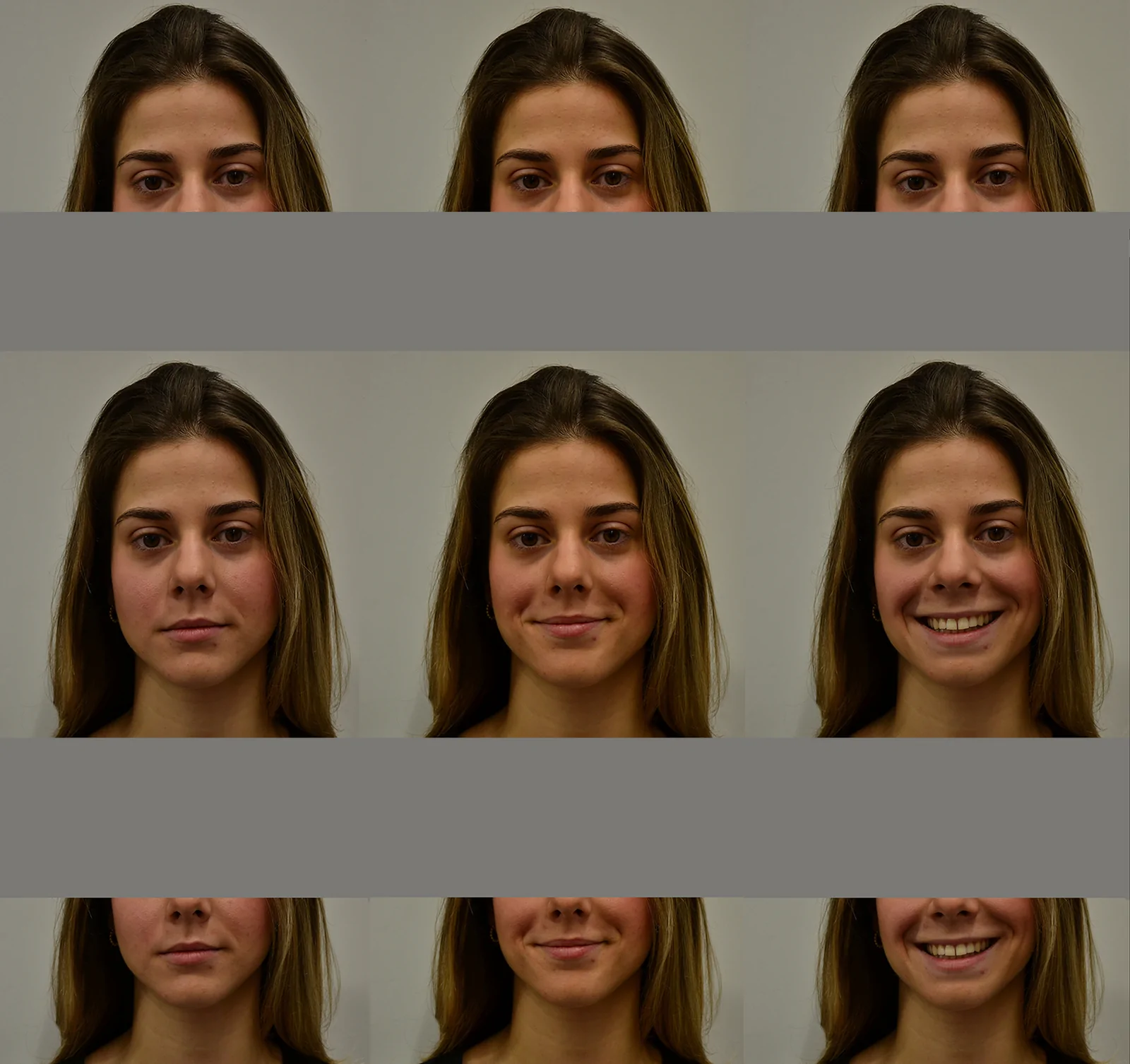
Sample of the stimuli adopted in the experiment derived from three photos of one person. In the bottom are displayed the mouth with three different expressions (neutral, slight smile, smile), in the top the neutral eyes expression (the same in the three images), in the middle the whole faces derived from the combination of the three mouths and the neutral eyes. In the middle row all the upper halves of the faces are identical, while the lower halves display three different mouth expressions.

In order to ensure the ecological validity of the results, we decided to use photographs of real people rather than faces generated by AI methods. We took 60 photographs of the faces of 20 Caucasian individuals of various ages and genders, making sure that all the pictures were taken in the same place, on a neutral background and with the same lighting to ensure consistency of conditions. Each person was photographed with three different facial expressions: neutral, with a hint of a smile and with a full smile. We asked each of them to agree to allow us to use their photographs for scientific research. Informed consent was obtained by the person depicted in Fig. 4 to publish her image in an online open access publication.

Using Adobe Photoshop CC 2019, for each person photographed we combined the three lower halves of each person’s face with the identical upper face displaying the neutral expression. In this way, we created 60 whole-face images that were only different in the lower part. All the photos were edited using Photoshop’s level masks method in the mouth region and showed no sign of manipulation, looking perfectly realistic.

In addition, we covered the upper part of each of the 60 photos (3 for each person) and the lower part from the 20 photos of the neutral expressions with a grey rectangle (Fig. 4). Overall, the stimuli consisted of 60 whole faces, 60 displaying the lower part of the face (neutral, with a mild smile, with a full smile, mouths) and 20 the upper part (neutral eyes).

The stimuli were presented on a computer MacBook Air 13″. Participants sat at a distance of about 50 cm from the screen, the background of which was grey. The images were 9.5 cm wide by 10 cm high.

#### Procedure

At the beginning of the experiment, it was explained to the participants that the study aimed to evaluate the visual characteristics of the images, and it was emphasized that there were no right or wrong answers, in order to reduce pressure and allow spontaneous and neutral responses.

The experiment was divided into three blocks, with the order of trials randomized within each block. In the first block, consisting of 60 trials, participants were shown in a sequence the 60 photos of the whole faces and asked to rate for each the perceived luminosity of the eyes (“How luminous/light up are the eyes in the photo?” on a 5-point scale ranging from “Not at all” (1) to “Very much” (5)). The rating scale values 1 to 5 and their corresponding labels (1 = not at all, 5 = very much) were displayed below each image.

Immediately after the participants had rated a photo the image disappeared, and the screen was left blank for 2000 ms before presenting the next one. There was no time limit for providing the answer.

In the second block (20 trials), participants rated the perceived luminosity of the 20 only-eyes stimuli taken from the photos with a neutral expression, using the same scale as in block 1.

Lastly, in the third block (60 trials), participants rated the perceived smiling of the mouth (“How smiling is this mouth?”) in the 60 only-mouth stimuli taken from the whole faces (lower part), on a 5-point scale ranging from “Not at all” (1) to “Very much” (5). The order in which the blocks were presented remained constant so that participants could not guess that the eyes of the 60 faces were actually three times fewer in number and always had the same expression.

At the beginning of each block, instructions that were readable on the screen informed the participants that they would be presented with single images showing whole faces or parts of faces and that their task was to provide an answer along a 5-point scale by pressing the keys on the keyboard. Four trial stimuli were presented before starting the experiment to familiarise participants with the task.

Stimuli presentation and ratings recording were carried out by the software PsychoPy (v2024.2.4).

Overall, the experiment lasted approximately 20 min. The procedure used in this and the other experiment reported in this paper were approved by the Ethics Committee for Research in the Human and Social Sciences—CAREUS of the University of Siena (Prot. n. 0,153,612). Data and research material are available on OSF at the following link: https://osf.io/dnvy6/?view_only=2c855e23a3f741c3894c59efd92c3116. This study’s design and its analysis were not pre-registered.

### Results

#### Perceived mouth expression

The ratings of the perceived smiling of the mouth in the cut-out lower parts of the photos (block 3) were analysed with a Linear Mixed-effects Model (LMM), including mouth expression as a fixed effect (sum coding) and participant and person represented in the photo as random effects (random intercept for both effects, and random slope for the effect of mouth for participants).

**Fig. 5:**
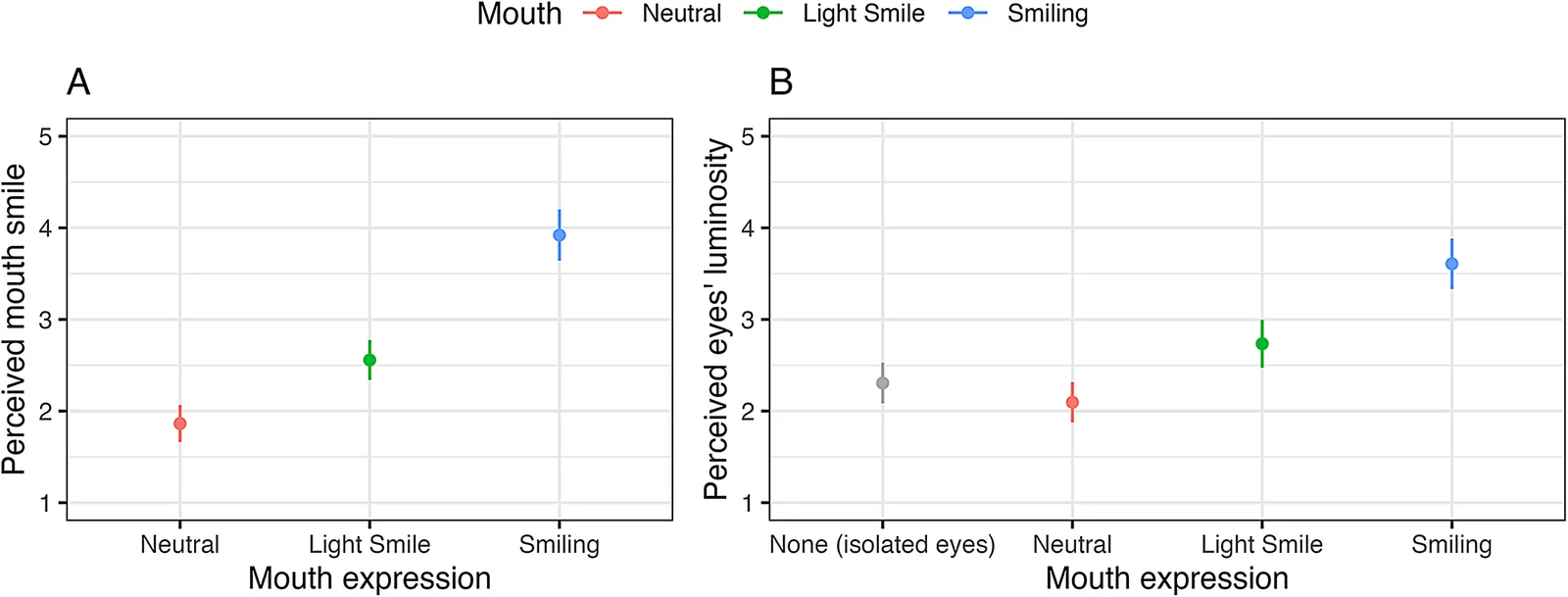
Plots of the average ratings in Experiment 1 as a function of mouth expression. **A**. Average ratings for the perceived smiling of the mouth (isolated) as a function of mouth expression. **B**. Average ratings for the perceived eyes luminosity in the whole face stimuli. Error bars are confidence intervals for the means based on standard errors in the LMM.

The results showed a significant effect of mouth expression on the ratings (*F*(2, 39) = 111.32, *p* < 0.001), and the pattern of means for the smiling ratings (Fig. 5A) confirmed that manipulation of the mouth expression worked: the neutral mouth had lowest ratings, significantly lower than the light smiling mouth (*p* < 0.001), which in turn had significantly lower ratings than the fully smiling mouth (*p* < 0.001).

#### Perceived eyes’ luminosity (isolated eyes)

**Fig. 6:**
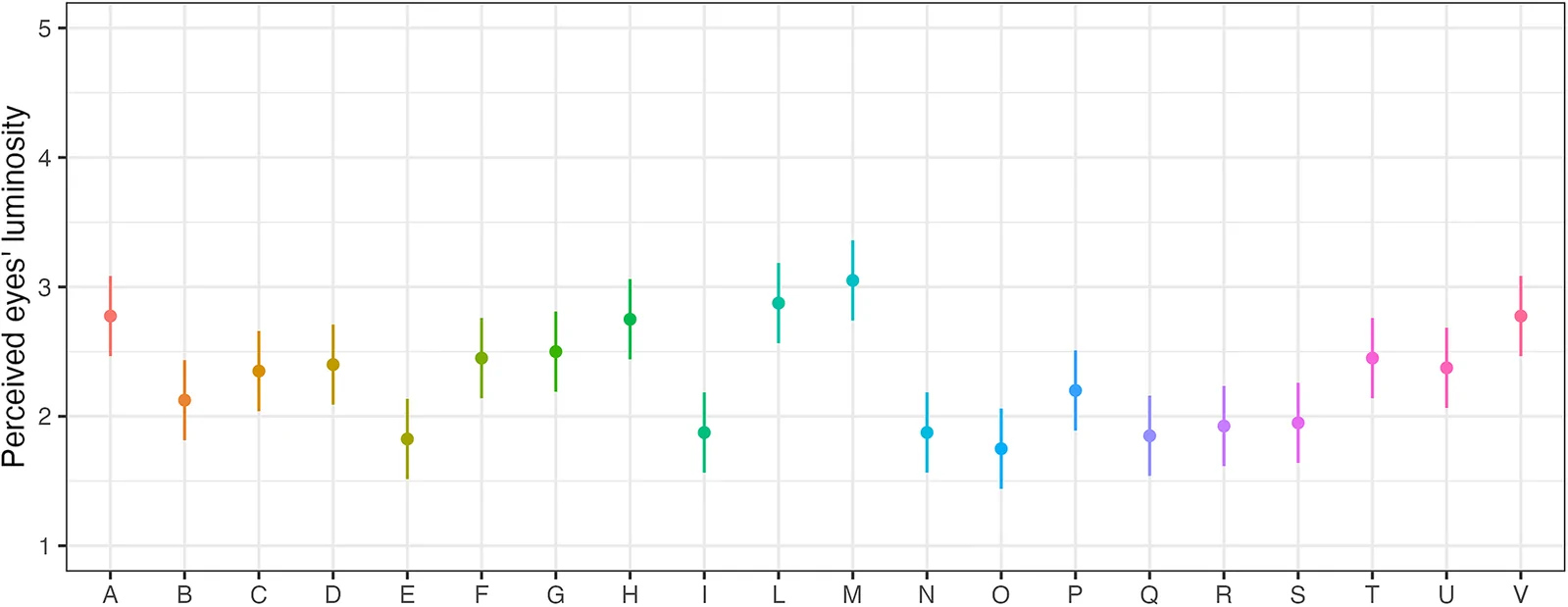
Plots of the average ratings for the perceived luminosity of the eyes presented by masking the lower part of the face, in Experiment 1 (block 2). Error bars are confidence intervals for the means based on standard errors in the LMM.

The ratings of the perceived eyes’ luminosity in the cut-out eyes from the photos of the neutral faces were analysed with an LMM, using the person in the photo as a fixed factor and participant as random factor (intercept). The results showed significant differences in the luminosity of the eyes of the different persons represented in the photos with a neutral expression (Fig. 6). The average ratings for the different faces, however, were in most cases lower than the midpoint of the scale, and the grand mean of ratings (across subjects represented and participants) was 2.31 (95% CI 2.14 – 2.48), indicating that the eyes were not seen very bright when isolated.

#### Mouth expression and perceived eye luminosity

The ratings of the perceived eyes’ luminosity in the whole face photos were analysed with a mixed-effects model, using the same fixed and random effects as in the analysis of the smiling ratings. The results showed again a significant effect of mouth expression on the ratings (*F*(2, 38.99) = 61.71, *p* < 0.001). As shown by the plots of the means in Fig. 5B, perceived eyes’ luminosity ratings varied in the same way as the perceived mouth smiling ratings. There were significant differences across all conditions in the pairwise comparisons (all *p* < 0.001), and significant linear (*Ψ* = 1.51, *p* < 0.001) and quadratic trends (*Ψ* = 0.23, *p* = 0.041) were exhibited.

To estimate the effect of the mouth on the perceived eyes’ luminosity, we conducted further analyses with LMMs. We predicted (in separate models) the eyes’ luminosity ratings from: (1) the ratings of the isolated eyes’ luminosity, (2) the ratings of the mouth smiling expression, and (3) both types of ratings. The participant and the person in the photo were included as random factors (intercept). The results showed that both the mouth smiling ratings and the isolated eyes’ luminosity ratings significantly predicted the eyes’ luminosity ratings in the whole face block (*B*_smile_ = 0.53, *B*_lumin_ = 0.22, all *p* < 0.001), both individually and when inserted simultaneously as predictors (*B*_smile_ = 0.52, *B*_lumin_ = 0.17, all *p* < 0.001). Mouth smile ratings alone explained 32.3% of the variance in the eyes’ luminosity ratings, and the ratings of the luminosity of the isolated eyes explained 3.4% of the variance. When in a further analysis the different types of ratings were allowed to interact (after centering), the results showed a significant interaction among the predictors (*t*(2378.5) =  − 4.80, *p* < 0.001). Interestingly the estimated interaction coefficient was negative (*B* =  − 0.06, 95% CI  − 0.09 to  − 0.04), indicating that the positive effect of the mouth smiling was slightly stronger for eyes that looked less bright when isolated.

Lastly, for each participant we computed the difference between the ratings of the eyes’ luminosity for the whole images and the ratings for the isolated eyes’ luminosity. This difference, representing the change in the isolated eyes’ perceived luminosity induced by the context, was analysed again with an LMM, which found a significant effect of the mouth expression (*F*(2, 46.25) = 61.58, *p* < 0.001), and the same positive trend in the means. Interestingly, the results showed that the perceived eyes’ luminosity in the context of a face with a neutral mouth expression (no smile) was slightly, but significantly, lower than the luminosity of the isolated eyes, as indicated by the mean ratings for the difference score that for the neutral mouth condition was negative and significantly different from 0 (*M* =  − 0.21, 95% CI  − 0.365 to  − 0.055).

#### Objective image properties

To rule out the possibility that the effect of the mouth on the ratings of the eyes’ luminosity could be due to differences in the objective image properties of the eyes induced by changes in the global context (*i.e*., when the mouth was changed), we conducted several additional tests. We first computed for each whole face stimulus a series of image properties relative to the region of the images containing the eyes (ROI). Specifically, we computed contrast^55^, energy^55^, Shannon’s entropy^56^, and saliency^57^ for the stimuli converted into grayscale, and luminance in different color spaces (RGB, CIELAB, YCbCr) for the original color stimuli. We then tested whether each of these metrics changes significantly as a function of the expression of the mouth, using a series of LMMs, including mouth expression as fixed effect, and face as random intercept. The results showed that the expression of the mouth did not affect significantly the saliency of the eyes (*p* = 0.103), or any of the objective measures computed on the grayscale (all *p* > . 59) or the color images (luminance RGB: *p* = 0.52; CIELAB: *p* = 0.51; YCbCr: *p* = 0.52). The full set of results and the code used to compute and analyse the metrics is reported in the supplementary materials.

### Discussion

**Fig. 7:**
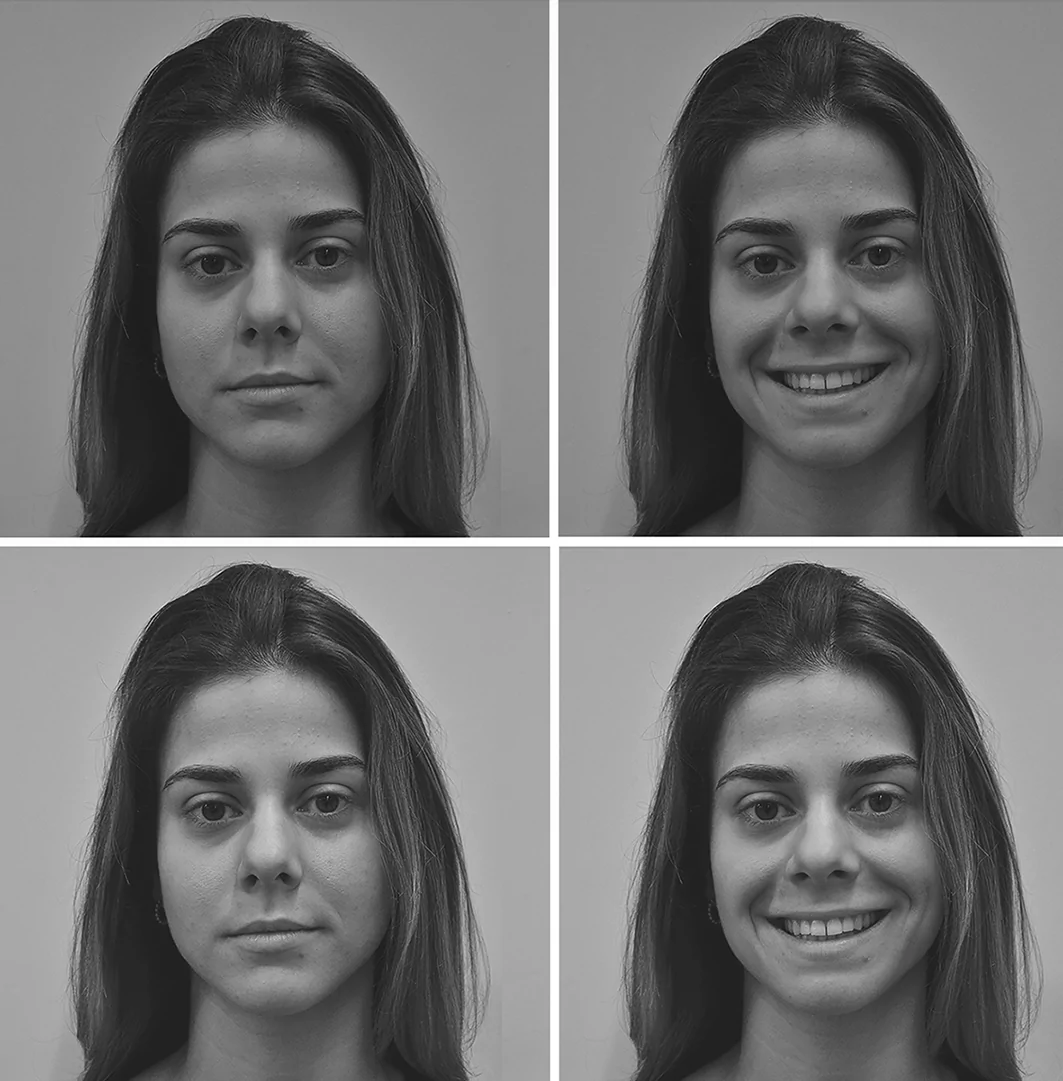
Achromatic versions (at two different luminance levels) of the image stimuli displayed in Fig. 4, for the neutral and smiling conditions.

The results of the analyses are consistent with the hypothesis suggested by the image reported in Mangini and Biederman^3^, that the smile could affect the luminosity of the gaze. Although the eyes remained unchanged, participants rated them as brighter when they were combined with a light or with a full smile. The effect, moreover, is not specific to achromatic blurred images, but it is also effective with highly ecological common photographic portraits. We intentionally chose to investigate this phenomenon by starting with a wide range of real faces displaying heterogeneous features, in order to establish its extent and to rule out the validity of the effect being limited to achromatic conditions. Indeed, there is no reason to suppose that the effect would not be present even with achromatic figures. A demonstration of this can be seen in Fig. 7. Subsequent studies could explore the magnitude of the effect using achromatic images and by manipulating different levels of luminance and contrast. Also, it would be interesting to investigate how chromatic cues and chromatic interactions (e.g., lip redness, iris colour, skin tone etc.) might modulate this effect of perceived eye brightness via chromatic-luminance interactions.

As suggested by some authors^11–13^ smiling is a crucial element in decoding facial emotions and the mouth plays a fundamental role in integrating the emotional information provided by the eyes. From a phenomenological point of view, it has been observed that neutral eyes, such as Mona Lisa’s ambiguous gaze, appear ‘to smile’ when combined with a smiling mouth^22^. In addition to these findings, the first experiment suggests that a smiling mouth not only alters the whole emotional expression of the face, but changes the appearance of the eyes, making them appear more luminous, vivid and alive, as spontaneously reported by some participants at the end of the experiment. Incidentally, none of the participants suspected that the rated images were composite faces with only neutral eyes. Even when the faces are juxtaposed, as can be observed in Figs. 4 and 7, the upper parts do not look identical, which in our view highlights the compelling nature of the effect.

Furthermore, the results of the first experiment indicate that this interaction between mouth and eyes’ luminosity is dependent on the perceived intensity of the smile. Crucially, while the mouth’s expression exerts a dominant influence, the effect remains anchored to the baseline evaluation of the eyes in isolation. Even the presence of the neutral mouth slightly affects the luminosity of the eyes, which appear less luminous than when isolated. Thus, this contextual effect seems to exhibit varying degrees of intensity instead of being an *all-or-nothing* categorical effect^15,16^. The effect is remarkably robust: it occurs in all stimuli and it is unaffected by participants’ gender or age, although it appears slightly stronger for eyes that are perceived as less luminous in isolation.

These results support the hypothesis that the brightness and vividness of the eyes might be modulated by the expression of the mouth. As several authors have argued, the influence of the smile on the interpretation of eye emotion is generally expected to occur unidirectionally, i.e., from a smiling mouth to neutral eyes. This effect has been explained as being due to the visual saliency of the smile, which allows for more extrafoveal projection^11,12,22^.

In order to further our comprehension of this phenomenon, the next experiment has been designed to explore whether the mouth-eye brightening effect only emerges when the eyes exhibit a neutral expression, or whether it can also occur when the eyes are genuinely smiling and the mouth is neutral. More specifically, we wondered whether a neutral mouth could ‘switch off’ the perception of smiling-luminous eyes when viewed in isolation, or whether the bright expression of Duchenne-featured eyes would remain unaffected by the mouth. If so, this would demonstrate a deeper interaction between the mouth and eyes than was previously thought.

## Experiment 2

Since Darwin’s classic work^47^ based on Guillaume Duchenne electrophysiological investigations in 1862, it is well known that genuinely smiling eyes display specific features based on the muscular actions involved in smiling, such as eye constriction and wrinkled eye corners^1,2,58,59^. Nevertheless, the results of the first experiment suggest that—in composite faces—the perceptual system tries to mitigate the intrinsic incongruity of the non-Duchenne smile by ‘extending’ the smile from the mouth to the eyes.

We wondered, therefore, what might happen if objectively smiling eyes were combined with a neutral mouth, beyond the plausible change in expression (e.g.,^18^). Would a neutral mouth correspondingly attenuate the perceived brightness of the eyes in the same way that a smiling mouth appears to enhance or ‘light up’ neutral eyes? To investigate this possibility, we merged the lower parts of neutral and smiling faces with the upper parts of the same faces showing smiling and neutral eyes, allowing us to compare each composite configuration with its respective individual baseline. For the control condition, participants were asked to evaluate also the brightness and the smiling impression of the eye-only area of the faces.

### Methods

#### Participants

Forty persons, different from those of the first experiment, participated in the experiment on a voluntary basis. They were aged from 18 and 75 years (M = 32, SD = 14). 47.5% of them were women (N = 19) and 52.5% were men (N = 21). The participants were all of Italian nationality and Caucasian ethnicity. All participants were naïve to the purpose of the experiment, and gave written informed consent according to the Declaration of Helsinki prior to their inclusion in the experiment. They all reported to have normal or corrected-to-normal visual acuity. A power analysis showed that for the design we used this sample size was sufficient to detect a medium main effect with > 90% power.

#### Experimental design and stimuli

**Fig. 8:**
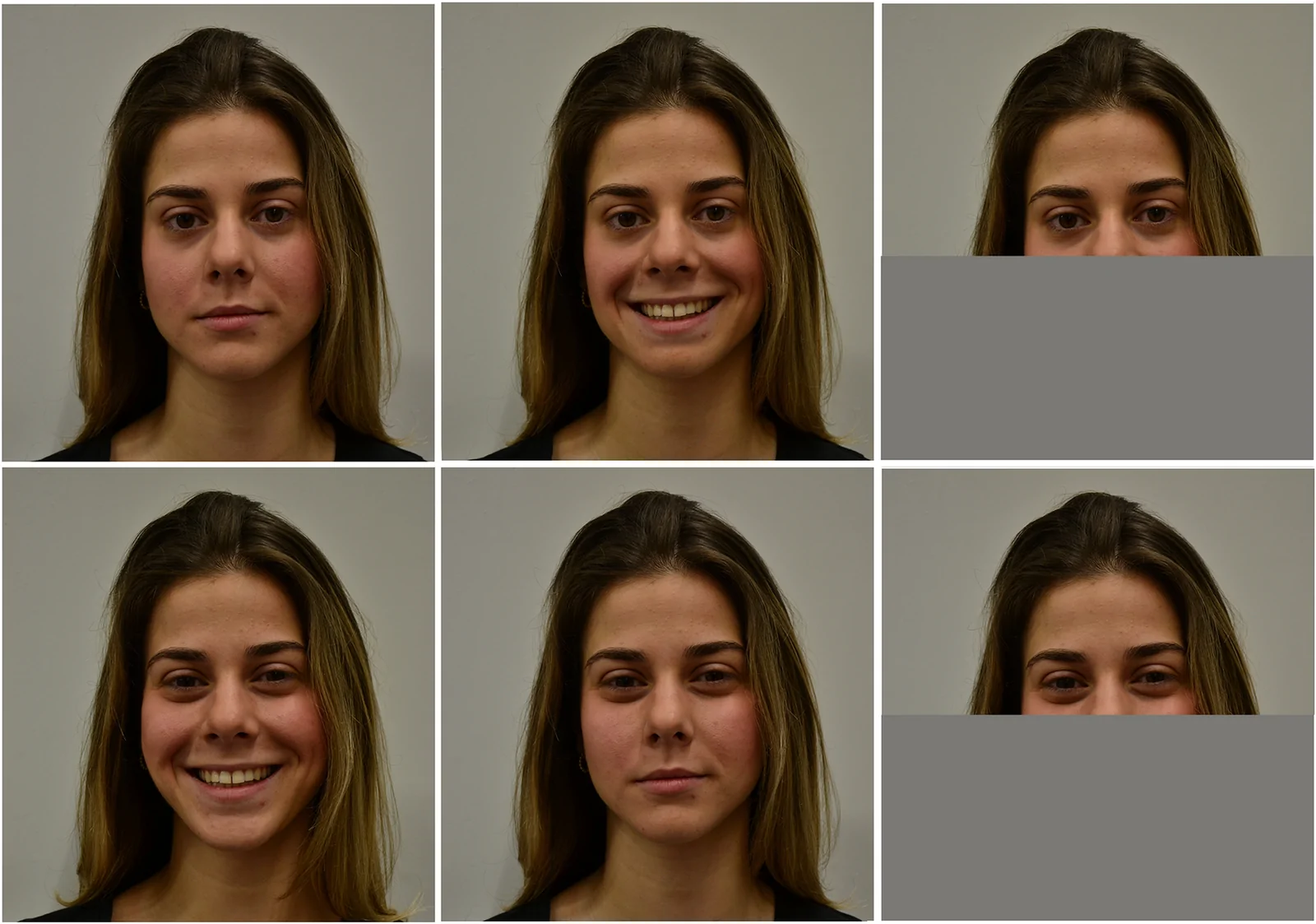
Sample of the stimuli adopted in the experiment derived from two photos of one person, one neutral and one smiling (top and bottom image on the left). Other two photos were generated combining neutral eyes with the smiling mouths (top image in the middle) and the smiling eyes with the neutral mouths (bottom image in the middle). On the right the only-eyes condition, neutral in the top and smiling in the bottom of the image.

The experiment had a factorial design with 2 factors manipulated within-participants: the expression of the mouth (*none* vs *neutral* vs *smiling*) and the expression of the eyes (*neutral* vs *smiling*). The stimuli were generated from a subset of the 60 photos of 20 men and women that was the basis for creating the stimuli used in Experiment 1. For experiment 2 we used 40 photos in which the models either smiled (20) or had a neutral expression (20) and generated further 40 images combining the smiling eyes with the neutral mouths (20) and the neutral eyes with the smiling mouths (20). In this way we had 80 stimuli, 20 for each of the four experimental conditions. In addition to these stimuli, we used the top halves of faces from 40 smiling and neutral photos. We covered the lower part of each image with a grey rectangular area, as in the first experiment (see Fig. 8).

#### Procedure

As in Experiment 1, participants were initially informed about the nature of the study and were asked to specify their age and gender, and then started the first of 3 blocks of trials.

In the first block participants were shown the 80 whole face stimuli in a random sequence and asked for each face to rate the perceived eyes’ luminosity/lighting up on a 5-point scale (same as in Experiment 1, block 1). In the second block the 40 cut-out eyes were presented in random order, and were rated again for perceived luminosity. In the third block the isolated eyes were again presented (in random order) but participants were asked to rate how much each eye looked smiling (on a 5-point scale, from “Not at all” to “Very much”). There was no time limit for providing the answer.

To ensure that participants did not infer the composite nature of the faces or the experimental hypotheses, the order of the blocks was fixed. On average, participants completed the experiment in about 15 min.

### Results

#### Perceived eyes’ luminosity

We analysed the perceived eyes’ luminosity ratings using linear mixed-effects models, including the eyes’ expression (2 levels: *neutral* or *smiling*), the mouth’s expression (3 levels: *neutral*, *smiling* or *none*—for the isolated eyes) and their interactions as fixed effects, and participant and person in the photo as random effects (random intercept for both factors, and random participant-level slopes for all the factors). Sum coding was used for the fixed effects.

**Fig. 9:**
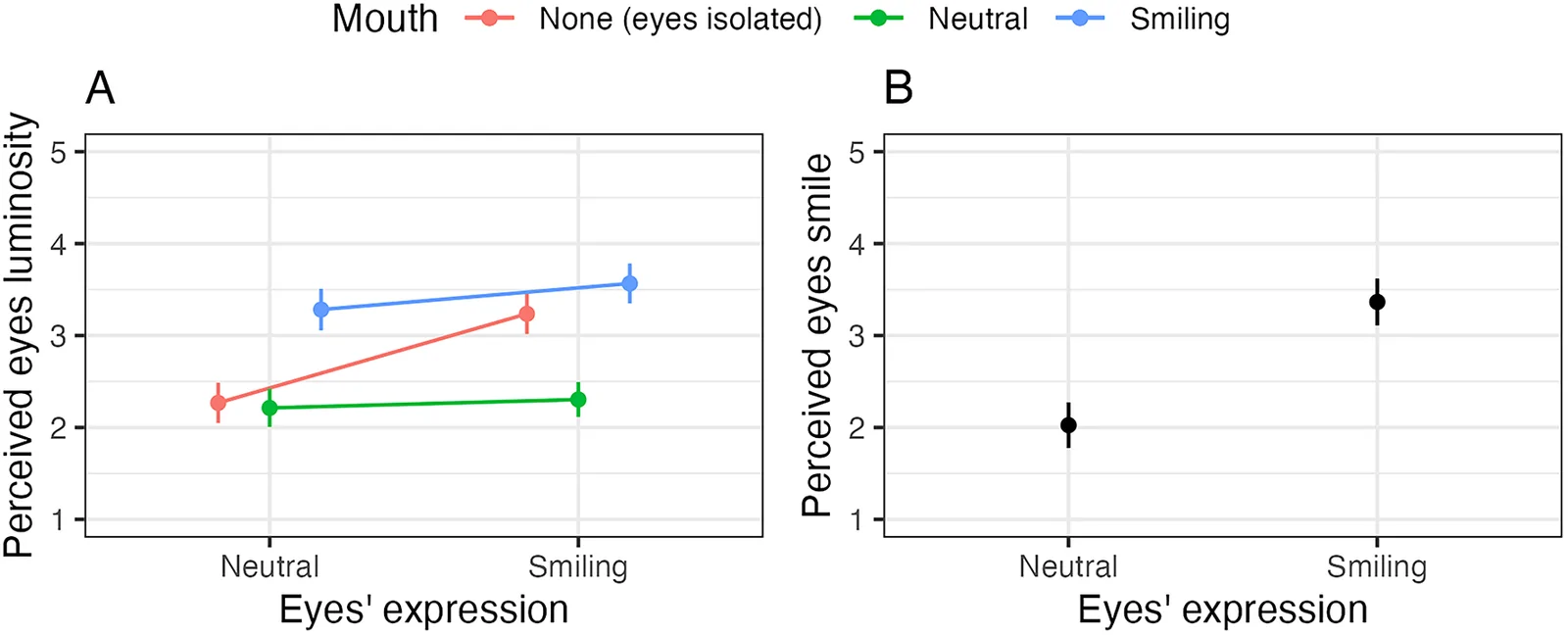
(**A**) Plots of the average ratings for the perceived eyes luminosity in Experiment 2 as a function of mouth and eyes expression. (**B**) Average ratings for the perceived smiling of the eyes (isolated) as a function of the eyes’ expression. Error bars are confidence intervals for the means based on standard errors in the LMM.

The results showed a significant main effect of eyes’ expression (*F*(1, 39.12) = 50.8, *p* < 0.001), a significant main effect of mouth’s expression (*F*(2, 39.06) = 57.7, *p* < 0.001) and a significant interaction between eyes’ expression and mouth’s expression (*F*(2, 67.86) = 28.5, *p* < 0.001). The average eyes luminosity ratings are plotted in Fig. 9A as a function of eyes’ and mouth’s expression.

Pairwise comparisons following the main effect of the eyes’ expression showed that the smiling eyes (*M* = 3.04) appeared significantly brighter than the neutral eyes (*M* = 2.59), averaging across the mouth’s expressions. Pairwise comparisons following the main effect of the mouth’s expression showed that, averaging across eyes’ expressions, the eyes appeared the least bright when the mouth’s expression was neutral (*M* = 2.26), significantly less bright than both the isolated eyes (*M* = 2.75, *z* =  − 7.85, *p* < 0.001) and the eyes in the context of a smiling mouth (*M* = 3.42, *z* = − 10.74, *p* < 0.001), which were rated as the brightest.

The analysis of the interaction showed that the effect of the eyes’ expression on their perceived luminance was strongly modulated by the context and dominated by the effect of the mouth’s expression. As it can be seen in the interaction plot in Fig. 9A, in fact, when the eyes were presented in a face with a neutral mouth expression the eyes’ smile had only a small (but significant) positive effect on the perceived luminance (*z* = 1.977, *p* = 0.048), the mean luminosity ratings were low both when the eyes had a neutral expression (*M* = 2.21) and when they had a smiling expression (*M* = 2.30).

When the mouth was smiling, instead, the both the neutral (*M* = 3.28) and the smiling (*M* = 3.57) eyes appeared significantly brighter than when the same eyes were presented with a neutral mouth (all *p* < 0.001), or isolated (neutral eyes: *M* = 2.27, *z *= 9.309, *p* < 0.001, smiling eyes: *M* = 3.24, *z* = 4.682, *p* < 0.001). With a smiling mouth the effect of the eyes’ expression was highly significant (*Ψ* = 0.28, *z* = 5.236, *p* < 0.001), and stronger than with a neutral mouth (*Ψ* = 0.09), but not as strong as it was for the isolated eyes (*Ψ* = 0.97, *z* = 7.545, *p* < 0.001), which was the condition in which the effect of the eyes’ expression was strongest. With neutral eyes the average ratings in the neutral mouth conditions were not significantly different from those in the isolated eyes (no mouth) conditions (*Ψ* = 0.05, *z* = 0.869, *p* = 0.660), conversely from what was found in the first experiment. With a smiling mouth, instead, the ratings for the luminosity of the smiling eyes were significantly higher than the ratings for the same eyes presented in isolation (*Ψ* = 0.33, *z* = 4.682, *p* < 0.001).

#### Perceived smiling of the eyes

We then analysed with LMMs the ratings of the perceived smile of the isolated eyes (block 3) as a function of eyes’ expression. We included random intercepts for participant and face and a random participant-level slope for the effect of eyes’ expression. The results showed a significant effect of the eyes’ expression (*F*(1, 39) = 68.32, *p* < 0.001), with smiling eyes (*M* = 3.37) being rated as significantly more smiling than neutral (*M* = 2.02) eyes (Fig. 9B).

Lastly, we examined the multilevel correlations between the ratings of the luminosity and of smiling of the isolated eyes, treating both participant and person in the photo as random factors. The results showed a significant, moderate correlation among the ratings (*r* = 0.57, *p* < 0.001).

#### Objective image properties

As we did for Experiment 1, we tested with LMMs whether objective image properties, computed relatively to the eyes region, varied as a function of eyes and mouth smile. As in Experiment 1, the results did not reveal a significant effect of the mouth on the metrics computed in the grayscale images (contrast: *p* = 0.48, energy: *p* = 97, entropy: *p* = 0.36, salience: *p* = 0.71) or on the luminance in the different color spaces (RGB: *p* = 0.192, CIELAB: *p* = 0.194, YCbCr: *p* = 0.192). Concerning the effect of the eyes, no significant effect was found on contrast (*p* = 0.68), energy (*p* = 0.095), entropy (*p* = 0.38) or saliency (*p* = 0.14). However, for all the luminance measures computed in the color version of the stimuli in the different color spaces (RGB, CIELAB, YCbCr) the results showed a significant effect of the eyes (all *p* < 0.01), with significantly higher values recorded for the smiling than for the neutral eyes. No significant interaction effect was found on any of the image properties considered (all *p* > 0.11).

### Discussion

The main results further support our hypothesis about the power of the mouth to modulate the apparent brightness of the eyes, even in different emotional facial expressions than smiling. Just as a smiling mouth significantly increased the perceived luminosity of neutral eyes, confirming the findings from the first experiment, a neutral mouth weakened the perceived brightness of smiling eyes. In other words, the same smiling eyes that—due to the characteristics of a Duchenne smile—are still judged as bright and luminous when the mouth area is removed, seem somehow ‘dimmed’ when combined with a neutral mouth.

Although eyes that genuinely smile appear more luminous than neutral eyes in both neutral and smiling mouth conditions, and are also physically different in terms of objective luminance, the subjective impression of luminosity can be significantly *increased* or *decreased* depending on the mouth’s expression. Even Duchenne smiling eyes exhibit a significantly different degree of apparent luminosity when viewed in combination with a smiling mouth than when viewed alone, confirming the graded nature of the effect. As well as in the previous experiment, results were obtained with a large set of different faces, balanced for gender, and treating faces as random stimuli, and thus should be generalizable beyond the stimuli set.

## General discussion

Previous research in face emotion recognition highlighted the role of the mouth in influencing the recognition of eyes’ emotion, particularly showing that a smiling mouth can bias the interpretation of neutral eyes toward happiness^8,10–13^. In this study, we approached the phenomenon from a gestalt and visual illusion perspective, inspired by the observation reported by Mangini and Biederman^3^. Our findings suggest that neutral eyes ‘light up’ and appear more vivid when combined with a smiling mouth, whereas smiling and luminous eyes could appear less bright when combined with a neutral mouth.

Crucially, a key finding of our study is that eye luminosity is not perceived as an all-or-nothing phenomenon or a binary categorical label (e.g., ‘bright’ vs ‘dim’) but as a graded perceptual feature.

As Experiment 1 demonstrated, the intensity of this effect varies in a dimensional way with the perceived degree of the mouth’s smile, while remaining sensitive to the perceptual baseline of the eyes (i.e., the ratings of the eyes presented in isolation). This distinction is crucial for understanding the nature of the effect. Our LMM analyses show that this baseline remains a significant predictor of the ratings, even when a smiling mouth is present. This suggests that the mouth’s expression might act as a contextual modulator of the intrinsic visual quality of the gaze.

These findings address the question of whether ‘luminosity’ is a distinct perceptual quality or a secondary evaluative projection of the positive affect induced by a smile. While a more parsimonious interpretation might suggest a global shift based on perceived happiness, the fact that ratings remain anchored to the eyes’ perceptual baseline (Experiments 1 and 2) argues against a mere affective projection. If the effect were purely evaluative, the subtle differences in the appearance of the individual eyes would likely be obscured by the smile’s influence; instead, the mouth context appears to modulate the perceived intensity of inherent perceptual properties of the gaze.

Moreover, the influence of the mouth expression on luminosity ratings is not limited to neutral eyes—which might be considered more ambiguous and susceptible to contextual bias—but significantly modulates the apparent brightness of smiling eyes (Exp. 2).

Furthermore, we observed a significant ‘inverse effect’: when eyes were paired with a neutral mouth, they were rated as significantly less bright compared to their isolated baseline. Notably, this ‘dimming’ effect occurred not only for neutral eyes (Exp. 1; Fig. 5B) but also for eyes originally extracted from a smiling face (Exp. 2; Fig. 9A), which were perceived as highly luminous even in isolation. This downward modulation suggests that the facial context can shift perception in both directions, providing support to the hypothesis that the phenomenon is a relational process rather than a simple additive affective projection, where a positive expression would only be expected to enhance, rather than suppress, the perceived intensity of a feature.

We acknowledge potential limitations inherent to the psychophysical task. The use of subjective ratings may introduce unseen response biases. Although participants typically did not suspect that the eyes were physically identical across conditions, demand or expectancy effects cannot be completely ruled out. However, the robustness and consistency of these ratings, coupled with the fact that objective image properties remained constant across conditions, reinforces the idea that we are measuring a consistent perceptual shift rather than low-level luminance changes. Further research using objective psychophysical measures (e.g., brief exposure paradigms, inversion, misalignment, or 2AFC brightness-matching tasks) will be necessary to clarify the extent to which the phenomenon reflects early perceptual processing or later interpretative appraisal. Moreover, developing more indirect or implicit paradigms, building upon the evidence provided by the current findings, will help refine the measurement of this subtle effect. Specifically, while concurrent measures of perceived happiness, attractiveness, or trustworthiness could establish whether ‘luminosity’ can be statistically dissociated from broader affective appraisals, from a gestalt perspective such a dissociation might be artificial. If luminosity is an emergent property of the facial ‘whole’, it constitutes a quality inherently inseparable from the expressive meaning of the face.

Taken together, these results support the interpretation of the effect as a compelling visual illusion grounded in the gestalt principle of *faceness*: a functional whole where the facial context influences the perception of individual features^27,33^.

From a theoretical standpoint, our findings could be compatible with the explanation proposed by Calvo and Nummenmaa^17^, according to which the morphological features and face configuration are rapidly processed by the perceptual system, followed by affective encoding and semantic categorisation. However, taking a more phenomenologically oriented approach, we hypothesise that eye brightness and emotional expression are inherently linked perceptual properties, emerging from the holistic facial configuration. Just as the *Wollaston effect* and the *composite face illusion* demonstrate, identical eyes can appear differently depending on the surrounding facial context^27,33^. From a gestalt perspective, emotional meaning is not something added by higher-level inferential processes to the visual configuration, but it is intrinsically connected to the pattern as it appears—an emergent property (see^60^). Consequently, eye luminosity in this paradigm should be regarded as an expressive-relational property rather than a mere surface luminance. Phenomenologically experienced as an *inner glow*, it is strictly tied to the impression of *aliveness* typical of the human gaze. To use an analogy, a red surface in a green context is perceived as being more saturated due to chromatic contrast, but also as being more salient and ‘vibrant’.

This intrinsic link imposes methodological considerations: because brightness and expression are inseparable, it is impossible to define a control condition in which the mouth is physically smile-like but does not affect perceived emotion. Consequently, the phenomenon is best studied within a theoretical framework that acknowledges the richness and relational nature of visual perception^51,53^. In recent years, the proposal that perception is far more sophisticated than traditionally assumed, involving rich contents that go beyond low-level properties—such as color, shape or size -, has been widely discussed and empirically supported by a growing body of research. For instance, many works explored the role of perceptual relations across several domains, such as causal perception, emotional and social relations, or face expressions, suggesting that such relations are inherently perceptual in nature and implicate automatic visual processing (see^51–54,61,62^). The present experiments provide clear signatures of this relational processing: identical eyes are perceived differently depending on the mouth context, affecting both perceived emotional meaning and ratings of luminosity. This effect is compelling, automatic, and persists even when images are juxtaposed for direct comparison—a hallmark of informational encapsulation (see^51–54,61^). Despite knowing that the upper parts of the faces are identical, we cannot help but *see* that when the mouth changes the eyes appear different.

While the border between perception, cognition, and emotion remains a subject of ongoing debate^63,64^, the surprise shown by observers when told the eyes are identical strongly suggests a process that is primarily perceptual rather than inferential. The uniqueness of ‘eye luminosity’ lies in its nature as a perceptual signature of the gaze, signaling facial aliveness. It is a distinctive and non-reducible perceptual quality that belongs to the domain of visual appearance, yet remains inherently relational as it is functionally dependent on the facial configuration. Specifically, it cannot be decoupled from the expressive configuration of the face, appearing as a quality that is inseparable from, yet not identical to, the broader expressive meaning.

In conclusion, this framework suggests that the mouth context triggers a genuine visual illusion, making the ‘lighting up’ phenomenological evidence rather than a post-perceptual cognitive judgment.

### How far does the ‘mouth-eye brightness’ interaction extend?

To explore the generality of this effect, we tested schematic and pareidolic faces. Recent studies, in fact, suggest the existence of a shared mechanism for facial expression in human faces and face pareidolia^28,65^.

**Fig. 10:**
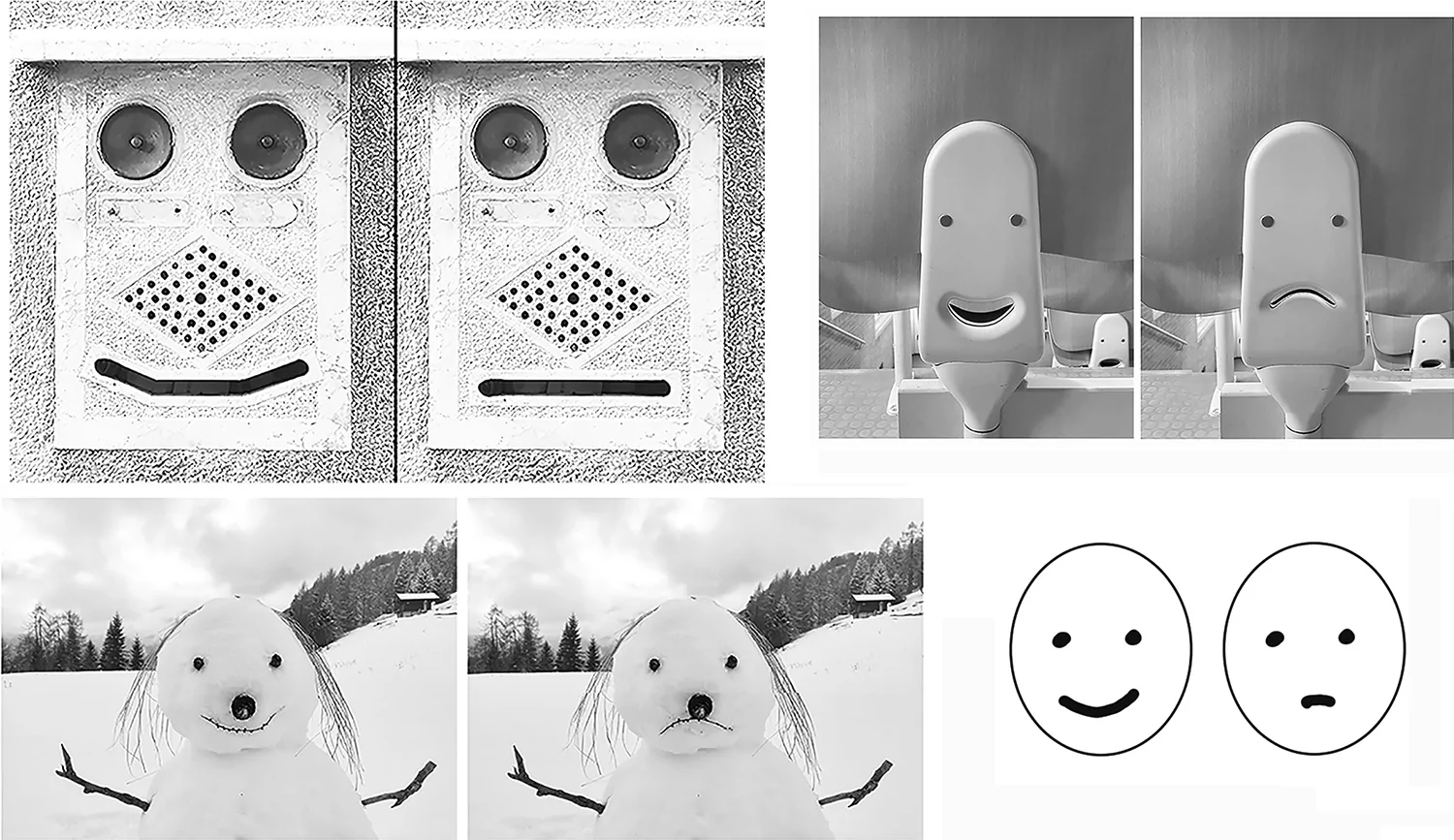
The effect of mouth orientation on pareidolic or schematic composite faces. Although the upper half is always the same, the parts that assume the function of ‘eyes’ appear more luminous and alive when the mouth smiles (pictures taken and merged with Photoshop by GP).

In the following examples, we manipulated a few different pareidolic or schematic faces by changing only the shape of the mouth. Remarkably, even in these simplified or non-human configurations, altering only the mouth caused the ‘eyes’ to appear brighter and more alive (Fig. 10). This indicates that the effect is a broad perceptual grouping phenomenon, not confined to naturalistic human faces.

The observation of this effect in pareidolic and schematic configurations provides further evidence against a purely cognitive or evaluative account. Unlike human faces, these stimuli lack complex social and biological attributes such as health or trustworthiness. Therefore, the fact that ‘eyes’ in inanimate objects appear to ‘light up’ when a smile-like shape is added suggests that the phenomenon is rooted in perceptual grouping and gestalt completion. This reinforces the idea that eye luminosity is an emergent perceptual property of the facial configuration itself, rather than a late-stage inference derived from social appraisal.

The mouth-induced enhancement of eye brightness may also explain previous observations in aesthetic evaluations of photographic portraits, where subtle manipulation of the mouth towards the smile increases aesthetic quality of the image and even the perceived attractiveness of the faces^45^.

**Fig. 11:**
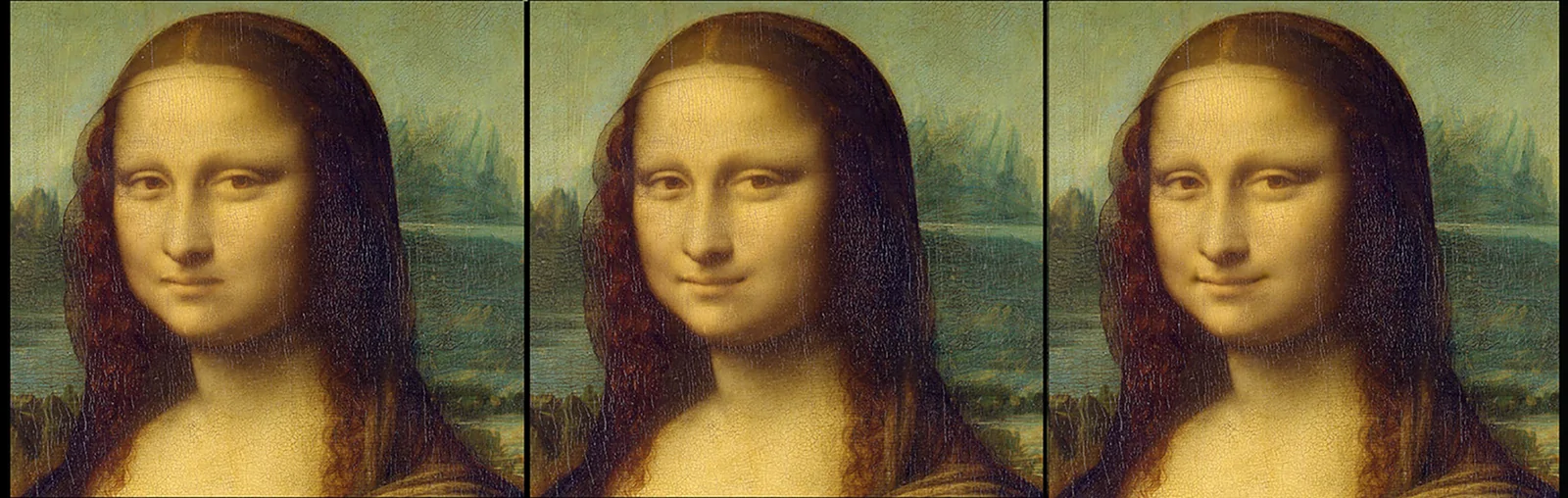
Slight changes to the mouth of the Mona Lisa affect the expression and, perhaps, enhance the luminosity of the eyes [the original in the middle, Leonardo da Vinci, c. 1505]. *Source*: https://www.wikiart.org/en/leonardo-da-vinci/mona-lisa.

The presence of the effect might be a relevant ingredient even in understanding the most iconic artwork, that is the Mona Lisa. Let’s see how her ambiguous gaze changes if we slightly remove the peripheral sides of the mouth, or slightly lengthen the right side. As Kontsevich and Tyler^22^ already observed, a smiling mouth gives to the eyes of Mona Lisa a happy expression (see also^23^), but we might add, they might seem even more vivid and luminous (Fig. 11).

Leonardo might have noticed this effect. In his artwork, the line between the lips appears to have simply been extended to the right, as if it were a late addition, without altering the central shape of the lips—which should appear thinner when smiling.

Moreover, as Leonardo might have equally realized, a shining smile in the eyes, which is only indirectly induced by the mouth, is aesthetically more appealing than a directly explicit Duchenne smile, involving the shape of the eyes^60^. Plausibly the smile – obtained by the elongation of the line between the lips and the *sfumato* around the mouth -, allowed in the Mona Lisa portrait her subtle and ambiguous, dynamically fluctuating, expression, as a result of perceptual organization factors^24–26^.

## Conclusions

This study suggests that the mouth context induces a subtle yet powerful visual effect on the gaze: a smiling mouth enhances the perceived brightness of the eyes, while a neutral mouth tends to diminish it. Crucially, this bidirectional modulation occurs regardless of the eyes’ initial state, affecting both neutral eyes (Exp. 1) and those already perceived as inherently luminous and smiling in isolation (Exp. 2). This effect reflects the holistic, relational nature of face perception, where facial parts are interdependent and the visual system integrates their configuration to generate emergent perceptual properties.

Why might the mouth be so effective? From a phylogenetic perspective, humans have evolved high sensitivity to faces and emotional expressions due to their adaptive social significance^53^. Anatomical interconnections of facial muscles, as highlighted by Darwin^47^, likely contribute to this effect: muscular structures of the mouth and eyes are functionally linked, facilitating coordinated facial expressions that can be perceptually amplified.

In his chapter on the *expression of joy*, for example, Darwin emphasised the key role played by the “intimate connection, as explained in the chapter on weeping, between the orbiculars, especially the lower ones, and some of the muscles running to the upper lip” (p. 203).

Building upon this biological foundation, we suggest that variations in mouth curvature and eye luminosity are not processed as decoupled features; rather, they are perceived as the unified, integrated expression of a single coherent emotion. We argue that the visual system inherently seeks a harmonious and consistent solution: when a person expresses a genuine emotion through a smile, the perception of enhanced eye luminosity complements the global configuration, ensuring that the entire face is perceived as a unified, living whole, where the eyes and mouth operate in reciprocal synergy to manifest a unitary expression.

Overall, these findings highlight that face perception is not only about recognizing individual features but also about emergent properties of the face as a whole, where the mouth context can modulate both emotional expression and the apparent luminosity and aliveness of the eyes. Even when the eyes appear inherently bright and vivid, a smile intensifies this radiance, whereas a neutral mouth can dampen it. This process maximizes facial expressivity and communicative value, ensuring that the gaze is perceived as truly ‘alive’. This insight opens new lines of investigations for understanding both perceptual mechanisms and aesthetic experience in faces.

## Data Availability

Data and research material are available on OSF at the following link: (https://osf.io/dnvy6/).
